# Effect of Melatonin on the stability and expression of reference genes in *Catharanthus roseus*

**DOI:** 10.1038/s41598-018-20474-2

**Published:** 2018-02-05

**Authors:** S. A. Sheshadri, M. J. Nishanth, V. Yamine, Bindu Simon

**Affiliations:** Phytoengineering Lab, School of Chemical and Biotechnology, SASTRA University, Thanjavur, India

## Abstract

The role of Melatonin in influencing diverse genes in plants has gained momentum in recent years and many reports have employed qRT-PCR for their quantification. Relative quantification of gene expression relies on accurate normalization of qRT-PCR data against a stably-expressing internal reference-gene. Although researchers have been using commonly available reference-genes to assess Melatonin-induced gene expression, but to-date, there have been no attempts to validate the reference-gene stability under Melatonin-supplementation *in planta*. In this study, we performed stability assessment of common reference-genes under Melatonin-supplementation and abiotic stress in leaves and seedlings of *Catharanthus roseus* using geNorm, NormFinder, BestKeeper, ΔCt and RefFinder algorithms. Nine candidate reference-genes were tested for stability in *C*. *roseus* (*FBOX*, *CACS*, *TIP*, *RSP9*, *EXP*, *EXPR*, *SAND*, *F17M5*, *ACT*) and our study inferred that while *EXP* and *EXPR* were the most-stable, *F17M5* was the lowest-stable gene in the leaf-fed samples. Among seedlings of *C*. *roseus*, *F17M5* and *TIP* were the most, while *ACT* was the least-stable gene. The suitability of selected stable reference-gene pairs was demonstrated by assessing the transcript levels of the Melatonin-biosynthesis gene *SNAT* under same conditions. Our study is the first to comprehensively analyze the stability of commonly-used reference-genes under Melatonin-induced conditions in *C*. *roseus*.

## Introduction

Melatonin, an indolic compound, initially identified as a neurohormone in bovine pineal gland is a ubiquitous molecule^1^ and it is found to have pleiotropic roles in diverse kingdoms^2,3^. It acts as an antioxidant, and has been widely used as a therapeutic compound to treat diseases like sleep disorders^4^, jet lag symptoms, convulsions^5^, aging and stress^6^, epileptic shock, alcohol-induced CNS damage and Alzheimer’s disease^7^. The presence of Melatonin across diverse species has been reported by several researchers^3,8^.

Melatonin in plants was discovered in 1995 and its potential roles are being widely studied in the recent decade. It is present endogenously in several commercial crops as well as food and beverages^9^. Though initially discovered as a potent antioxidant^10^, melatonin was found to have various beneficial properties in many plant varieties. Phytomelatonin was found to regulate plant growth^11^, photosynthetic efficiency^12^, delay senescence of leaves^13^ and control the defense mechanisms^14^. One of our studies indicated that exogenous supplementation of Melatonin to the medicinal plant *Catharanthus roseus* could enhance the therapeutic metabolite profile, thereby increasing its bio-reductant capacity^15^. Similar applications of Melatonin to commercial crops (like coffee and soybean) resulted in enhanced fatty acids content^16^ and improved profile of alkaloids^17^. Strikingly, Melatonin based physiological and molecular effects on plants was concentration dependent. Though Melatonin concentration up to 1000 µM has been tested^18,19^, but most reports suggest concentration below 100 µM to be more beneficial in plants^16,20,21^. At the molecular level, many scientific studies are being conducted to elucidate the role of Melatonin in regulating expression profiles of various genes across diverse plant genera. Recent reports have suggested that Melatonin supplementation could regulate genes involved in growth and development^22^, photosynthesis^16^, redox reactions^23^, abiotic stress tolerance (UV^24,25^, wounding and sucrose supplementation^26–30^), specialized metabolism (such as phenylpropanoid metabolism: Phenylalanine Ammonia Lyase [*PAL*], Chalcone synthase [*CHS*], Chalcone isomerase [*CHI*], Flavanone 3-hydroxylase [*F3H*], Dihydroflavonol reductase [*DFR*] Anthocyanidin synthase [*ANS*])^18^ and sucrose metabolism (Cell Wall Invertase [*CWIN*], Sucrose synthase [*SUSY*])^27–30^.

In order to accurately assess the gene-level changes caused by Melatonin *in planta*, qRT-PCR (Quantitative Real time PCR) is a handy tool preferred by researchers mainly due to its rapidity, sensitivity, specificity and reliability^20,31–33^. Relative analysis of gene expression is the most commonly used method in qRT-PCR, wherein the target gene expression is measured in relation to the standard reference gene^34,35^. Although several factors come into play to determine the accuracy of the results (like quality control, primer specificity and reaction parameters), the selection of the most stable reference genes is essential to obtain accurate normalization of qRT-PCR data^36–38^. Studies have shown that a few reference genes show subtle variation in their expression profile under different biotic or abiotic stress conditions^39,40^, which therefore implies that using single gene as an internal control for normalization of qRT-PCR data could lead to inappropriate interpretations^41^.

Stability analysis of the candidate reference genes commonly employs known algorithms like geNorm, NormFinder, BestKeeper, comparative ΔCt method and RefFinder^42–45^. The geNorm algorithm assigns stability values (M) to the candidate genes based on the logarithmically transformed expression ratio between two genes. The lower the stability value, higher is the stability of the selected gene. The minimum number of reference genes necessary to obtain accurate qRT-PCR data normalization is calculated by analyzing the pairwise variation (V) between the geometric mean of the logarithmically transformed expression values of the candidate genes^46^. Another algorithm, NormFinder ranks the genes based on stability values derived *via* comparison of the variations in gene expression present across different groups. The most stable genes are those with the lowest Stability Value (SV)^43^. BestKeeper algorithm^47^, ranks the candidate reference genes according to their coefficient of correlation (R), relying on the standard deviation (SD) and coefficient of Variance (CV)^44^. The ΔCt method assesses the stable genes among the given set of reference genes by comparing their relative expression levels. The RefFinder tool integrates the above mentioned algorithms and provides geometric mean values to obtain comprehensive ranking for all the candidate reference genes.

*C*. *roseus*, an immensely potent medicinal plant is being widely utilized for the overproduction of commercially important therapeutic metabolites like vincristine and vinblastine, known to be potent anticancer agents. The bioactive alkaloids from *C*. *roseus* have also been attributed with anti-diabetic, anti-hypertensive and disinfectant properties^48^. Recent reports have highlighted that Melatonin could augment therapeutic metabolites like rauwolscine, fisetin and 6-acetyl morphine in *C*. *roseus*^15^. Several reports have also pointed at the role of Melatonin in regulating these metabolites at gene expression level^18,20^. Among the Melatonin-based qRT-PCR studies in plants, most expression data have been normalized using genes like *TUB* (Tubulin; Soybean^16^), 16SrRNA (Arabidopsis and Tobacco^14^), Actin (Citrullus^48^), *EF1α* (Elongation factor-1α; Apple^21,32^); *UBI* (Ubiquitin; Rice^22,49^) and Cyclophilin (Arabidopsis^14^), without testing the stability of these reference genes under the specific Melatonin treated conditions.

In our study, we tested the stability of the reference genes under different Melatonin concentrations in leaf versus seedling, as well as in leaves exposed to different abiotic stress conditions (UV, wounding and sucrose). Based on the previous stability reports, we selected nine reference genes: *FBOX* (F-box domain containing protein), *CACS* (Clathrin adaptor complex subunit), *TIP* (TIP41-like protein), *RSP9* (40 S ribosomal protein S9), *EXP* (Expressed protein of unknown function), *EXPR* (Expressed protein of unknown function), *SAND* (Sand family protein), *F17M5* (unknown protein F17M5) and *ACT* (Actin). The stability of genes were assessed based on geNorm, NormFinder, BestKeeper, comparative ΔCt method and RefFinder. Finally, the most stable reference gene was used for normalizing the expression levels of Melatonin biosynthetic pathway gene, *SNAT* (serotonin N-acetyltransferase), in all the tested conditions.

## Results

### Experimental conditions and PCR amplification

The candidate reference genes (*FBOX*, *CACS*, *TIP41*, *RSP9*, *EXPR*, *SAND*, *F17M5*, *ACT* and *EXP*) were tested for stability under different concentrations of Melatonin (1, 10 and 300 µM; water was used as control) along with abiotic stresses like UV, sucrose supplementation and wound. The specificity of amplification using the primer pairs was confirmed by the appearance of single dissociation curve (Supplementary Figure 1) as well as single band on 1.5% agarose gel electrophoresis (Supplementary Figure 2). The amplification efficiencies were computed *via* LinRegPCR software and all efficiencies were found to be within the acceptable range of 90–105% (http://www.gene-quantification.de/real-time-pcr-guide-bio-rad.pdf). The reactions indicated good linear relationships, with R^2^ > 0.99 (Table 1).

**Table 1:** Details of genes and primers used and amplification specifications in qRT-PCR.

Gene symbol	Gene name	Accession ID	Primer sequence (5′-3′); Fwd//Rev	Amplicon size	T_m_	Efficiency (%)	R^2^	Reference
*FBOX*	F-box domain containing protein	At5g15710	TTGGGTTGAGATAAGTCGGATG// CTGGCTGTTGTATGATTGAAGAG	199	78.9	97	1.00	^44^
*CACS*	Clathrin adaptor complex subunit	At5g46630	GCGGCGATGTCCTCATCAATC// GCATCCTCCAATCTGACGAACTG	128	78.6	103	1.00	
*TIP41*	*TIP*41-like protein	At4g34270	AGGATGGAAGCAGGAAAGGT// ACCGCAATATGGTGTTGTGA	119	77.9	101	1.00	
*RSP9*	40S ribosomal protein S9	AJ749993	GATGGTGCACGTTTCCTTTT// TGGGTCCTTCTCATCCAAAG	208	80.7	94	1.00	
*EXPR*	Expressed protein (unknown function)	At5g12240	CGCATTCTCAACCTCTTCC// ATCACCACGGTCACTTCC	168	78.6	98	1.00	
*SAND*	*SAND* family protein	At2g28390	TTGACCCTGCTTCTCGTTCT// GCAAGCTGCTGATAGGTGAG	192	80.4	90	1.00	
*F17M5*	unknown protein *F17M5*	At4g33380	CGGCTTCCTCCTGAATGTC// GCTCATACGGGCAATAAACC	181	80.7	105	1.00	
*ACT*	Actin	DQ117850	CTATGTTCCCAGGTATTGCAGATAGA// GCTGCTTGGAGCCAAAGC	241	78.4	103	1.00	—
*EXP*	Expressed protein (unknown function)	At4g26410	ACAATACCATCGCCATCAC// AAGAGGACTGCTGGAAGG	172	82.2	103	1.00	^44^

### Expression levels of candidate genes

**Figure 1:**
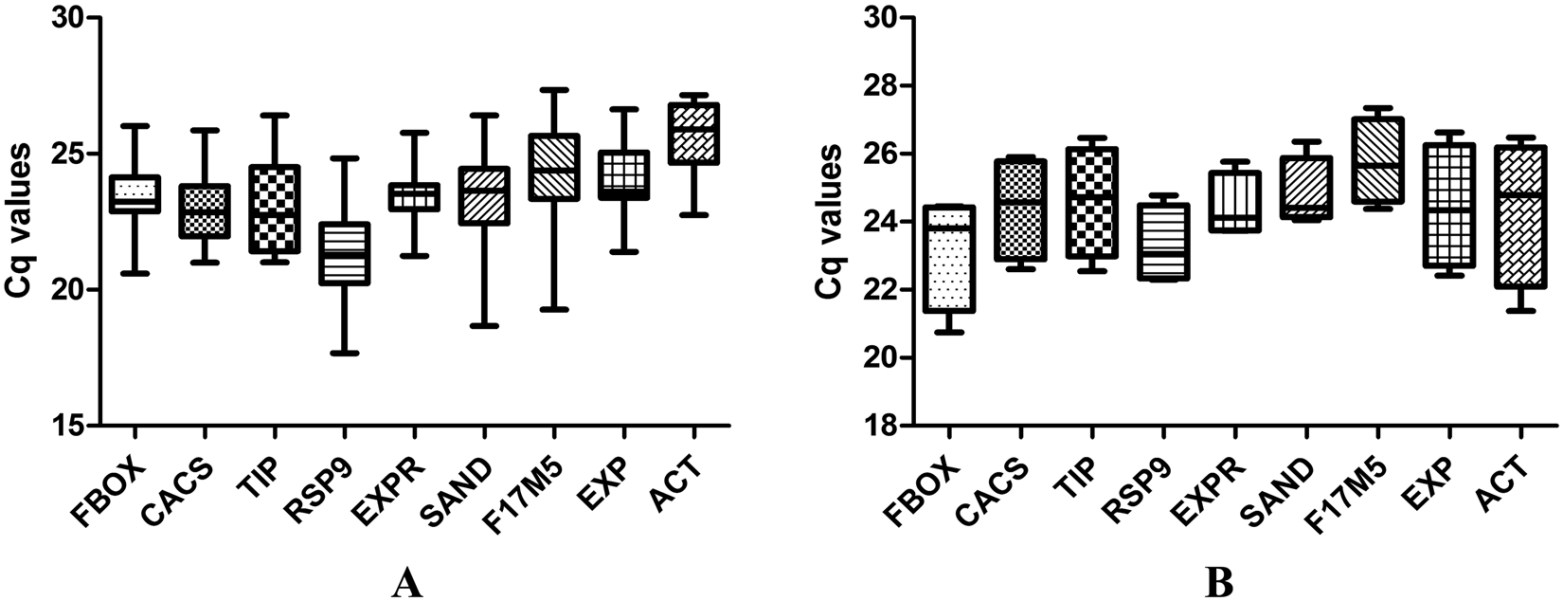
Cq values representing the expression levels of selected candidate reference genes in the (**A**) leaf-fed tissues and (**B**) seedlings of *C*. *roseus*. The central line in the boxes represent median, while whiskers represent minimum and maximum values.

The raw amplification data from qRT-PCR was used to obtain baseline evaluated Cq values *via* LinRegPCR software. The average expression levels (Cq ± SD) of candidate genes in leaf-fed tissues was between 21 and 26 cycles, and among the genes analyzed, *RSP9* was the highest (Cq = 21.382 ± 1.766), while *ACT* was the lowest expressed gene (Cq = 25.539 ± 1.351). *EXPR* showed the least variation (~4 cycles) while *F17M5* was the highest (>5 cycles). In *C*. *roseus* seedlings, *FBOX* was the highest expressed (Cq = 23.204 ± 1.723) while *F17M5* was the lowest expressed gene (Cq = 25.755 ± 1.258). The least variation was shown by *EXPR* (~2 cycles) and highest by *ACT* (~5 cycles). The Cq values have been represented in a box-and-whiskers plot (Fig. 1A,B).

### Stability analysis of candidate genes

The stability analyses were carried out in two sets: leaf-fed and seedlings group. Since various concentrations of Melatonin were associated with diverse functions *in planta*, the stability analysis of the candidate reference genes was performed under three concentrations of Melatonin: 1, 10 and 300 µM (water-treated sample was used as control) under commonly studied abiotic stresses: UV, sucrose and wounding (no stress was used as control). The analysis employed four most commonly used algorithms: geNorm, NormFinder, BestKeeper, ∆Ct and the resulting stabilities across all algorithms were comprehensively ranked using RefFinder.

### geNorm analysis

**Figure 2:**
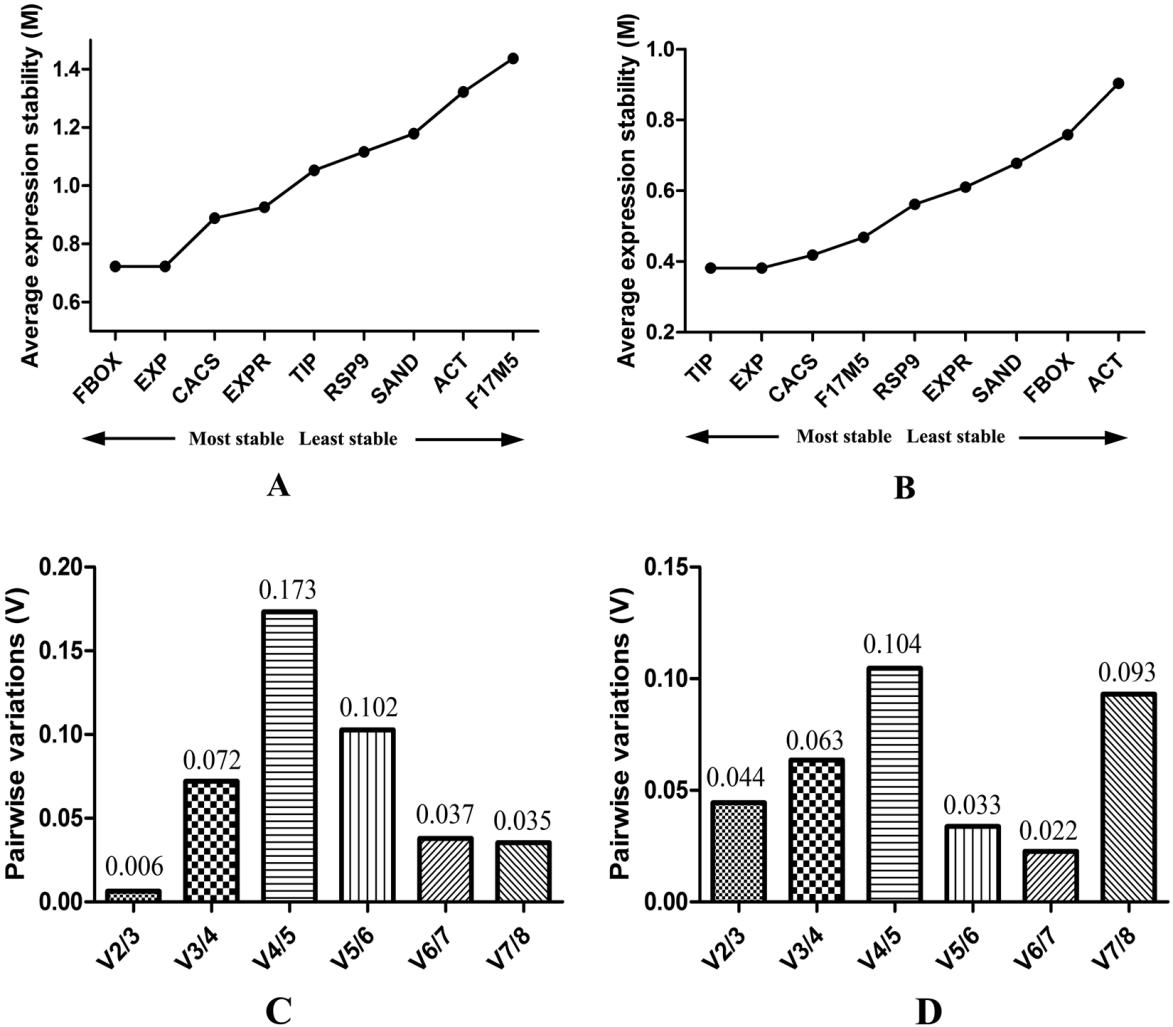
geNorm analysis of candidate reference genes for Melatonin-treated tissues in *C*. *roseus*: (**A**) leaf-fed samples and (**B**) seedlings. Pairwise variation analysis was performed to evaluate the minimum number of reference genes necessary for accurate data normalization in the *C*. *roseus*: (**C**) leaf-fed and (**D**) seedlings. The V2/3 values for all samples were below 0.15.

geNorm software ranks the candidate genes based on their stability values (M value). The analysis performed on *C*. *roseus* leaf-fed samples inferred that *FBOX* and *EXP* were the most stable (M = 0.723), while *F17M5* was the least stable candidate gene (M = 1.437; Fig. 2A). However, a differing trend was observed with the seedling samples, wherein *TIP* and *EXP* showed the highest stability (M = 0.382; Fig. 2B). Thus, based on the geNorm analysis for *C*. *roseus* samples, we inferred that *FBOX* and *EXP* were the best performing genes in the leaf-fed samples while *TIP* and *EXP* were the most stable genes in the seedlings group (Table 2). None of the selected genes were disqualified as all the M values were within the set limits.

**Table 2:** Compiled stability ranking of candidate reference genes in the leaf-fed tissues and seedlings of *C*. *roseus via* geNorm, NormFinder and ∆Ct methods.

Sample→	Leaf-fed tissues						Seedlings					
Genes	geNorm		Normfinder		∆Ct		geNorm		Normfinder		∆Ct	
	M value	Rank	Accumulated Standard Deviation	Rank	Average SD	Rank	M value	Rank	Accumulated Standard Deviation	Rank	Average SD	Rank
*EXP*	0.723	1	0.51	1	1.17	1	0.382	1	0.489	4	0.83	5
*FBOX*	0.723	1	0.965	5	1.38	5	0.758	8	0.721	7	0.95	7
*CACS*	0.889	3	0.624	3	1.23	3	0.418	3	0.198	2	0.71	2
*EXPR*	0.926	4	0.597	2	1.22	2	0.61	6	0.693	6	0.87	6
*TIP*	1.053	5	0.95	4	1.37	4	0.382	1	0.277	3	0.74	3
*RSP9*	1.116	6	1.075	7	1.45	6	0.562	5	0.57	5	0.82	4
*SAND*	1.179	7	1.073	6	1.48	7	0.678	7	1.038	8	1.1	8
*ACT*	1.322	8	1.522	8	1.79	8	0.904	9	1.367	9	1.42	9
*F17M5*	1.437	9	1.575	9	1.84	9	0.468	4	0.108	1	0.69	1

Also, a pairwise variation analysis was performed to evaluate the number of reference genes needed for precise normalization of gene expression based on the pairwise variation value (V). If the V_n_/V_n+1_ value falls below the threshold value of 0.15, it is assumed that ‘n’ number of genes is sufficient for accurate data normalization^46^. Since the V2/3 values for all the samples fell within the threshold value, it was inferred that only two reference genes were sufficient to normalize the qRT-PCR data (Fig. 2C,D).

### NormFinder analysis

**Figure 3:**
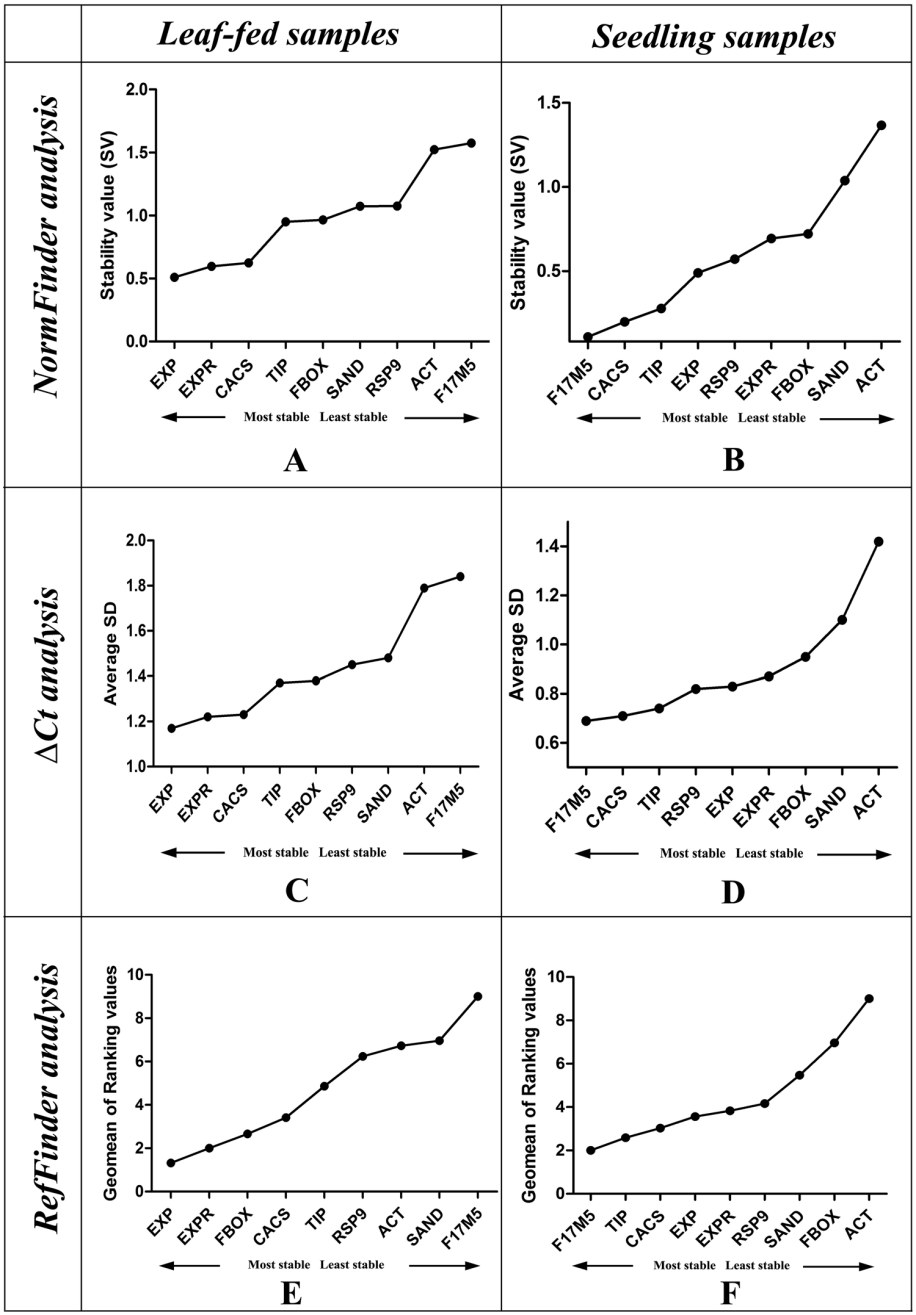
NormFinder analysis of candidate reference genes for Melatonin-treated tissues in *C*. *roseus*: (**A**) leaf-fed samples and (**B**) seedlings; ∆Ct analysis of candidate reference genes for Melatonin-treated tissues in *C*. *roseus*: (**C**) leaf-fed samples and (**D**) seedlings; RefFinder analysis of candidate reference genes for Melatonin-treated tissues in *C*. *roseus*: (**E**) leaf-fed samples and (**F**) seedlings.

The NormFinder software assigns ranks to the candidate genes based on their Stability Values (SV) and the results obtained were largely similar to those of geNorm analysis. Among the leaf-fed samples, *EXP* and *EXPR* displayed highest stability (SV = 0.51 and 0.597; Fig. 3A); while in the seedlings group, *F17M5* and *CACS* were the most stable ones (SV = 0.108 and 0.198 respectively; Fig. 3B). However, *F17M5* (SV = 1.575) and *ACT* (SV = 1.367) were the least stable genes in the leaf-fed and seedling group respectively (Table 2).

### Bestkeeper analysis

The algorithm employed by BestKeeper relies on the Standard Deviations (SD) and Pearson’s correlation coefficient (*r*) to rank the candidate reference genes^47^. The gene(s) with SD values above 1 were disqualified and rank was not assigned. The Bestkeeper results further confirmed the geNorm and NormFinder results, wherein, among the leaf-fed samples, *EXPR* and *FBOX* were the only two qualified genes (SD = 0.67 and 0.9 respectively), while in the seedlings group, *EXPR* and *SAND* were the most stable ones (SD = 0.67 and 0.77 respectively) among the four genes which qualified for the analysis. Although seven genes in the leaf-fed group and five genes from seedlings group were excluded from ranking, their SD values have been enlisted in Table 3.

**Table 3:** BestKeeper results for candidate reference genes in the leaf-fed tissues and seedlings of *C*. *roseus*.

Rank	Genes	Sample	n	std dev [+/−CP]	Geo Mean [CP]	AR Mean [CP]	Min [CP]	Max [CP]	CV [% CP]	min [x-fold]	max [x-fold]	SD [+/−x-fold]	Coeff. of corr. [r]	p-value
1	*EXPR*	Leaffed	16	0.67	23.44	23.46	21.25	25.77	2.84	−4.58	5.02	1.59	0.86	0.001
2	*FBOX*			0.9	23.29	23.33	20.59	26.02	3.84	−6.5	6.64	1.86	0.74	0.001
3	*EXP*			1.05	23.99	24.03	21.4	26.63	4.35	−6.05	6.21	2.06	0.903	0.001
4	*ACT*			1.1	25.5	25.54	22.75	27.15	4.32	−6.76	3.13	2.15	0.409	0.116
5	*CACS*			1.15	23.05	23.1	20.99	25.85	4.97	−4.18	6.96	2.22	0.898	0.001
6	*RSP9*			1.38	21.31	21.38	17.67	24.83	6.47	−12.52	11.48	2.61	0.84	0.001
7	*TIP*			1.4	22.96	23.02	21.01	26.4	6.06	−3.87	10.86	2.63	0.851	0.001
8	*SAND*			1.4	23.32	23.39	18.67	26.41	6	−25.02	8.53	2.65	0.886	0.001
9	*F17M5*			1.46	24.07	24.15	19.27	27.35	6.03	−27.74	9.72	2.74	0.725	0.001
1	*EXPR*	Seedlings	4	0.67	24.42	24.44	23.74	25.77	2.75	−1.61	2.54	1.59	0.902	0.098
2	*SAND*			0.77	24.79	24.81	24.05	26.35	3.11	−1.68	2.95	1.71	0.722	0.278
3	*RSP9*			0.91	23.27	23.29	22.3	24.77	3.92	−1.97	2.83	1.88	0.915	0.085
4	*F17M5*			0.93	25.73	25.76	24.37	27.35	3.62	−2.56	3.06	1.91	0.984	0.016
5	*TIP*			1.2	24.57	24.62	22.55	26.46	4.88	−4.08	3.7	2.3	0.991	0.009
6	*FBOX*			1.23	23.15	23.2	20.75	24.45	5.29	−5.3	2.46	2.34	0.93	0.07
7	*CACS*			1.23	24.38	24.41	22.6	25.9	5.05	−3.43	2.88	2.35	0.992	0.008
8	*EXP*			1.44	24.38	24.43	22.42	26.63	5.9	−3.89	4.76	2.72	0.994	0.006
9	*ACT*			1.54	24.28	24.36	21.37	26.47	6.34	−7.49	4.58	2.91	0.86	0.14

### ∆Ct analysis

The ∆Ct analysis assigns ranks to the candidate reference genes without involving the primer efficiencies into calculations and the results largely corresponded with the geNorm, NormFinder and BestKeeper results. While *EXP* and *EXPR* were the best stable genes in *C*. *roseus* leaf-fed samples, *F17M5* and *CACS* were the most stable ones in the seedlings group (Fig. 3C,D and Table 2).

### RefFinder analysis

The results obtained *via* the four algorithms: geNorm, NormFinder, BestKeeper and ∆Ct were integrated in order to yield a consolidated ranking for the candidate reference genes and this data can further be employed to validate/quantify target gene expression levels in *C*. *roseus* samples using similar feeding strategies. The results were expressed as geometric mean of ranking values and lower the value, better is its stability. We inferred that *EXP* and *EXPR* were the best performing genes in the leaf-fed samples (Fig. 3E) whereas *F17M5* and *TIP* were the most stable genes in the seedlings (Fig. 3F). The results have been summarized in Table 4.

**Table 4:** Consolidated stability ranking of candidate reference genes in *C*. *roseus via* RefFinder.

Leaf-fed tissues		Seedlings	
Genes	Geomean of ranking values	Genes	Geomean of ranking values
*EXP*	1.32	*F17M5*	2
*EXPR*	2	*TIP*	2.59
*FBOX*	2.66	*CACS*	3.03
*CACS*	3.41	*EXP*	3.56
*TIP*	4.86	*EXPR*	3.83
*RSP9*	6.24	*RSP9*	4.16
*ACT*	6.73	*SAND*	5.47
*SAND*	6.96	*FBOX*	6.96
*F17M5*	9	*ACT*	9

### Target gene validation

**Figure 4:**
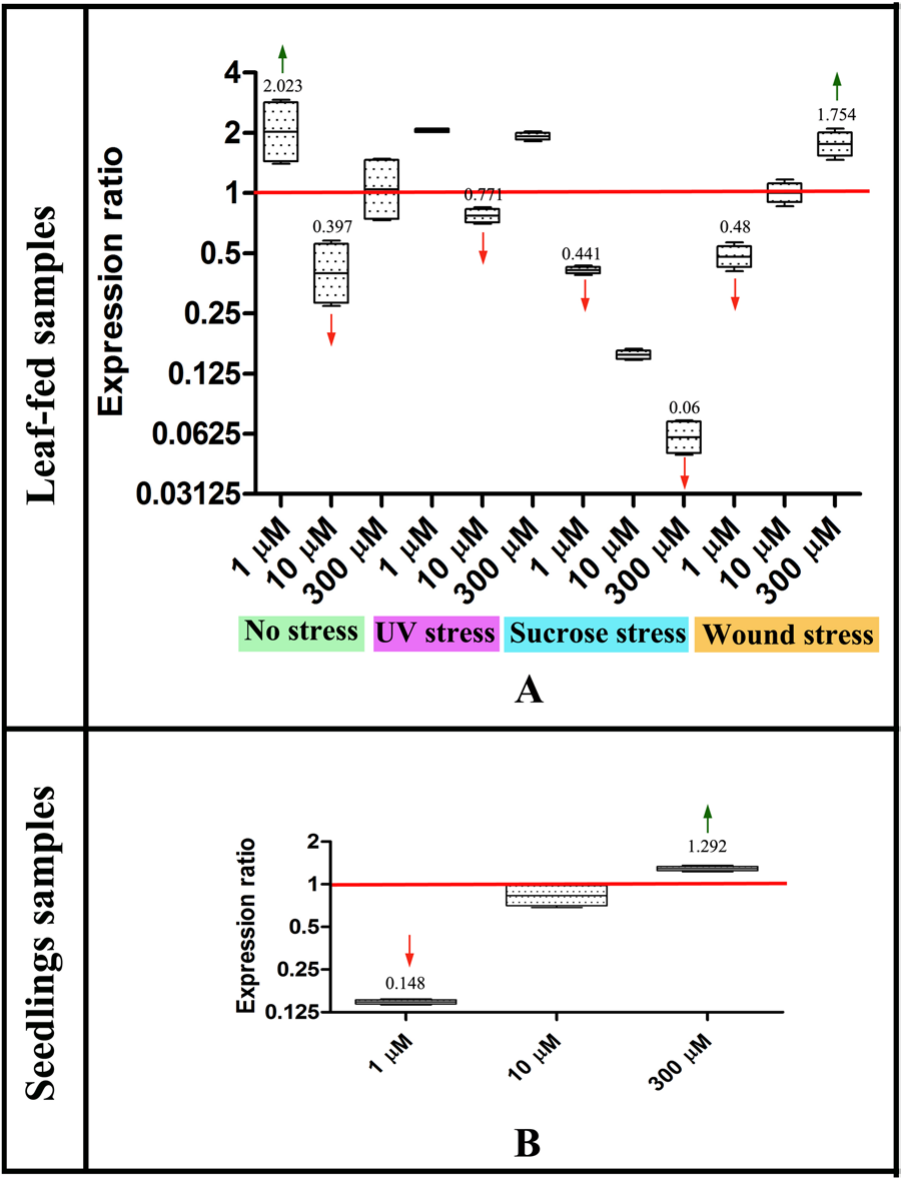
Relative expression levels of target gene SNAT using the most stable reference gene pairs (as determined by RefFinder analysis) in *C*. *roseus*: (**A**) leaf-fed samples normalized with the average expressions of *EXP* and *EXPR*; and (**B**) seedling samples normalized with the average expressions of *F17M5* and *TIP*. The values indicated represent significant fold change (as determined by REST software; p < 0.001). Green arrows indicate upregulation; red arrows represent downregulation.

One of the principal Melatonin biosynthesis genes, *SNAT* was used to validate the normalization performance of the best ranking reference genes. It catalyzes the conversion of Serotonin into N-acetyl-serotonin, which is one of the most important steps leading to Melatonin biosynthesis. The expression profile of *SNAT* from leaf-fed and seedling samples was performed using REST software. Although on an overall scale, Melatonin treatment caused downregulation of *SNAT*, the effects varied across different stress treatments. 1 µM Melatonin treatment to leaves was seen to upregulate *SNAT* in UV-treated and control groups, whereas the opposite trend was observed in sucrose supplemented and wound stressed samples (p < 0.05). Significantly, 10 µM treatment downregulated *SNAT* expression levels across all treatment groups (p < 0.05), barring wounding stress where no significant change in transcript levels was observed. On the other hand, 300 µM treatment caused upregulation in UV and wound stress, whereas it downregulated *SNAT* in the sucrose treated sample (p < 0.05). No significant fold change was observed in the control group (Fig. 4A). Among the seedling samples, although 1 µM and 10 µM samples significantly downregulated *SNAT*, 300 µM treatment to seedlings caused an upregulation (Fig. 4B).

## Discussion

Melatonin has been known for over two decades and ever since its discovery in plants, the number of reports available relating plant systems and Melatonin has been on the rise. Melatonin’s role in regulating specialized metabolic processes (in-turn regulating the defence responses) has been an extremely crucial area of study, and several reports have attempted to understand the underlying mechanisms therein. Studying gene expression profiles can help decipher the molecular-level implications of Melatonin supplementation to plant systems, and qRT-PCR is a very handy tool to assess the transcript levels *in-vitro*. One of the most followed strategies in gene expression analysis is relative quantification, wherein the levels of target genes are assessed in reference to a standard housekeeping gene, also called as reference gene. Some of the peer-reviewed articles studying the molecular level implications of Melatonin in plants have employed commonly used reference genes like *TUB*^16^, 16SrRNA^14^, *Actin*^49,50^, *EF1α*^21,32^ and *UBI5*^22,51^. Contrarily, certain reports have indicated that in model plants like *N*. *benthamiana*, *N tabacum* and *A*. *thaliana*, genes like *TUB* and *Actin* were associated with low stability^41–43^. Using an incorrect reference gene may lead to improper normalization of the qPCR values, thereby leading to inappropriate interpretations^42^. Owing to the ever-increasing studies being carried out to decipher the molecular role of Melatonin *in planta*, it was necessary to perform the validation of potential reference genes under Melatonin supplementation in presence and absence of abiotic stress conditions (UV, sucrose supplementation and wounding), since it is known that these conditions could potentially co-regulate defence (terpenoids indole alkaloids and Phenylpropanoid metabolism) as well as sucrose metabolism *in planta*^27–30^. This report, to the best of our knowledge, is the first study that has validated the stability of reference genes in the widely-known medicinal plant *C*. *roseus*, under Melatonin-induced conditions in presence of abiotic stress.

The selection of candidate genes was based on previously reported study and the gene-specific primers were designed accordingly. Nine candidate reference genes (*FBOX*, *CACS*, *TIP*41, *RSP9*, *EXPR*, *SAND*, *F17M5*, *ACT* and *EXP*) were selected to perform the stability analysis. *RSP9* and *ACT* were the most and least expressed genes in the leaf-fed samples, while among the seedlings, the corresponding genes were *FBOX* and *F17M5*. The stability assessment of the candidate genes was performed using four most widely used algorithms: geNorm, NormFinder, BestKeeper and ∆Ct. The results obtained through all the algorithms were considered in comprehensively ranking the best performing gene *via* RefFinder tool. Among the *C*. *roseus* leaf-fed samples, *EXP* and *EXPR* were inferred as the best performing stable reference genes among all treatment groups, while in the seedlings group, *F17M5* and *TIP* were the most stable ones. The differences observed in the pair of best ranked reference genes in the leaf-fed and seedlings group clearly indicate that the performance of candidate reference genes is tissue-specific. It was additionally observed that two reference genes were sufficient for accurate normalization of qRT-PCR data in *C*. *roseus* leaf-fed and seedling samples. Although there are reports studying the stability analysis of candidate reference genes in *C*. *roseus*^44,45^, Melatonin-based evaluation of stable reference genes has not been reported till date.

In order to validate the selected reference genes, the transcript levels of one of the principal Melatonin biosynthesis genes, *SNAT* was assessed in the leaf-fed and seedling samples of *C*. *roseus*. *SNAT* is a metabolic enzyme which catalyses the conversion of serotonin to N-acetylserotonin and is found to be influenced by various environmental stresses. The basis for selection of *SNAT* was its involvement in the regulation of melatonin levels during abiotic stress conditions^52,53^. Interestingly, 1 µM treatment to *C*. *roseus* leaf-fed and seedling tissues had conflicting effects, where the former showed an upregulation in *SNAT* level, while the latter indicated a downregulation. Although the relative expression of *SNAT* in the 300 µM treated tissues was almost similar among the leaf-fed tissues and seedlings, 10 µM treatment downregulated *SNAT* levels. Among the stressed tissues, sucrose stress completely downregulated the *SNAT* expression, while a concentration dependent effect was observed in the wounding stress group, wherein the *SNAT* levels gradually increased against increasing Melatonin concentration. The results didn’t indicate any trend in the UV stress treated samples, where 1 µM and 300 µM significantly upregulated, but 10 µM downregulated *SNAT* levels. This observation bears significance as it has been reported that under stressed conditions, the biosynthesis of Melatonin undergoes an increase, attributed primarily to the biosynthetic genes *SNAT* and *HIOMT/ASMT*, thereby increasing the tissue-Melatonin content^53^. However, under exogenous supply of Melatonin, the fate of *SNAT* still remains largely unknown. Our report is the first to assess the levels of *SNAT* under different concentration of Melatonin with induced abiotic stresses (UV, sucrose and wounding).

In summary, we have presented a comprehensive analysis of the stability of nine genes for the anti-cancerous medicinal plant *C*. *roseus* under exogenous Melatonin supplementation. Our study provides a reliable list of most stable reference genes, which could be a used by researchers for accurate normalization of expression patterns of genes in *C*. *roseus* under Melatonin treatment.

## Materials and Methods

### Plant material and Melatonin treatment

*C*. *roseus* seeds were purchased from PanAmerican Seeds and were surface sterilized with 75% alcohol for 1 minute, followed by 10% Clorox treatment for 10 minutes. Further, these seeds were blotted on sterile Whatman filter paper (no. 1) saturated with different concentrations of Melatonin (0, 1, 10 and 300 µM) and allowed to germinate under a 12:12 light:dark regime at 60% relative humidity. After germination, the seedlings were transferred to pots containing cocopeat under 12:12 light regime in a greenhouse, and supplemented every 24 hours with the respective Melatonin solutions for 15 days. These seedlings were then flash-frozen in liquid nitrogen and stored at −80 °C till RNA isolation.

The leaf feeding experiment was performed using 4–6 weeks old *C*. *roseus* leaves as per previously reported protocol^15^. The leaves were incised above the petiole region and placed in different concentrations of Melatonin (0, 1, 10 and 300 µM) solution for 5–7 days. The selection of the Melatonin concentrations was based on our previous experiments, where these selected concentrations showed significant physiological effects^15^.

### Stress treatment

The leaf-fed tissues of *C*. *roseus* were subjected to abiotic stresses (Ultraviolet radiation [UV], sucrose supplementation and wounding). UV stress was induced by placing the leaf-fed tissues under UV light (Philips TUV T8; 48 µW/cm^2^) for 2 minutes followed by incubation in the respective Melatonin solutions for 24 hours^27,54^. Sucrose supplementation was performed by placing the leaf-fed tissues in 90 mM sucrose solution followed by intermittent dabbing for 24 hours^30^. Wound stress was induced by making incisions in at least 50% of the leaf area using a surgical blade and further incubated in the respective Melatonin solutions for 24 hours^28^. The stressed tissues were then snap frozen in liquid Nitrogen and stored at −80 °C.

### RNA isolation and cDNA synthesis

The total RNA was extracted from *C*. *roseus* seedlings and leaves using NucleoSpin RNA plant kit (Macherey-Nagel, Germany) following the manufacturer’s instructions. The integrity of RNA was tested *via* gel electrophoresis and the A260:A280 quantitation was performed using Nanodrop spectrophotometer. The RNA samples with A260:A280 values between 1.9 and 2.1 and A260:A230 values higher than 2.0 were taken for further experiments. Total RNA quantified to 2.5 µg was digested with DNaseI (RNase free, Thermo Fisher Scientific) following the prescribed protocol. The first strand cDNA synthesis was performed using PrimeScript RT Reagent kit (Takara Bio, USA) following manufacturer’s protocol. All cDNA samples were stored at −20 °C till use and diluted 2.8-times using nuclease-free water prior to qRT-PCR analysis.

### Selection of candidate reference genes and qRT-PCR analysis

A total of nine candidate reference genes were selected in our study. Eight candidate reference genes: *FBOX*, *CACS*, *RSP9*, *TIP*, *EXPR*, *SAND*, *F17M5* and *EXP* were selected based on a previously reported stability data in *C*. *roseus*^44^, and their primers were obtained accordingly. In addition, the commonly employed reference gene *ACT* was also considered for the stability analysis and primer pair was designed using Primer3 software (http://bioinfo.ut.ee/primer3-0.4.0/). The details of the genes and their primers used are enlisted in Table 1.

For each primer pair, PCR reaction efficiency (E) was computed using LinRegPCR software from the non-baseline corrected amplification data (Table 1). The qRT-PCR was performed with ABI7500 Real Time PCR instrument using FastStart Essential DNA green MasterMix (Roche Diagnostics GmbH, Germany). One 7.5 µl reaction mixture comprised of 3.75 µl Mastermix, 1 µl of 1:2.8X diluted cDNA, 0.5 µl of each primer (5 pmol) and 1.75 µl nuclease free water. The PCR was carried out as per the following cycling parameters: 94 °C for 10 mins; 40 cycles of 94 °C for 30 s, 52 °C for 40 s, 72 °C for 30 s; and finally 72 °C for 5 mins. Dissociation curves were obtained between 60 °C and 95 °C. The specificity of the primer pairs was verified by the appearance of single peak in the dissociation curve analysis (Supplementary Figure 1) and additionally verified by appearance of single band on 1.5% agarose gel electrophoresis (Supplementary Figure 2). Three replicates were performed for each qRT-PCR reaction.

### Expression data and stability analysis

The expression of candidate reference genes were depicted in terms of Cq values (Fig. 1A,B). The genes were analyzed for stability using commonly used algorithms: geNorm, NormFinder, BestKeeper, ΔCt method and RefFinder. Through the geNorm analysis, the average pairwise variation is calculated for a particular gene in reference to all other selected genes, thereby generating the stability value (M). Lower the M-value, higher is the stability of the gene. The genes with M > 1.5 were excluded from ranking. Pairwise variation analysis was performed to estimate the minimum number of genes required for normalization^46^. NormFinder tool identifies stably expressing gene among groups and assigns a stability value (SV). BestKeeper algorithm performs stability analysis using statistical parameters like standard deviation (SD), Pearson’s correlation coefficient (*r*) and co-efficient of variance (CV). The genes having SD > 1 were not taken for analysis and ranking was assigned based on the SD values^44^. ΔCt method compares the relative expression of pairs of genes in the given sample. When the ΔCt value for a given pair is constant it is said that the genes are stably expressed^55^. Through this method a large set of genes can be compared against each other. RefFinder tool uses the data generated by all the four methods to give a comprehensive ranking to choose the most and least stable genes among the given set^56^.

### Expression of target gene

In order to validate the best-ranking reference genes obtained from all the algorithms, the expression profile of a selected target gene is studied. The primer for *SNAT* (Fwd- CCGCCATTACAAAATTCACC; Rev- GAATCCACAACAGAGCGTCA) was designed using Primer3 and the amplification specificity was observed *via* dissociation curve analysis (Supplementary Figure 1). The relative changes in the target genes in terms of relative expression ratio were calculated using Relative Expression Software Tool (REST) which uses The Pairwise Fixed Reallocation Randomisation Test^34^ to determine the significance of the result obtained and indicates whether the chosen reference genes are suitable for normalization. The expression results were adjusted according to the primer efficiency value using the formula:

$${\rm{Ratio}}={({{\rm{E}}}_{{\rm{target}}})}^{{\rm{\Delta }}\mathrm{Ct}{\rm{target}}(\mathrm{control} \mbox{-} \mathrm{sample})}/{({{\rm{E}}}_{{\rm{ref}}})}^{{\rm{\Delta }}\mathrm{Ct}{\rm{ref}}(\mathrm{control} \mbox{-} \mathrm{sample})}$$

## Electronic supplementary material

Supplementary Info file
