# Supplementary material for: Effect of Melatonin on the stability and expression of reference genes in Catharanthus roseus
Source: Sci Rep. 2018 Feb 5;8:2222. doi: 10.1038/s41598-018-20474-2 (PMC5799177; doi:10.1038/s41598-018-20474-2)

SUPPLEMENTARY INFORMATION FILE

Effect of Melatonin on the stability and expression of reference genes in *Catharanthus roseus*

S. A. Sheshadri[1], M. J. Nishanth[1], V. Yamine[1] and Bindu Simon[1*]

[1] Phytoengineering Lab, School of Chemical and Biotechnology, SASTRA University, Thanjavur, India
*** Correspondence:**
Dr. Bindu Simon
bindusimon@scbt.sastra.edu
Phone: +91-4362-264101

Supplementary Figure-1: Melting curve analysis

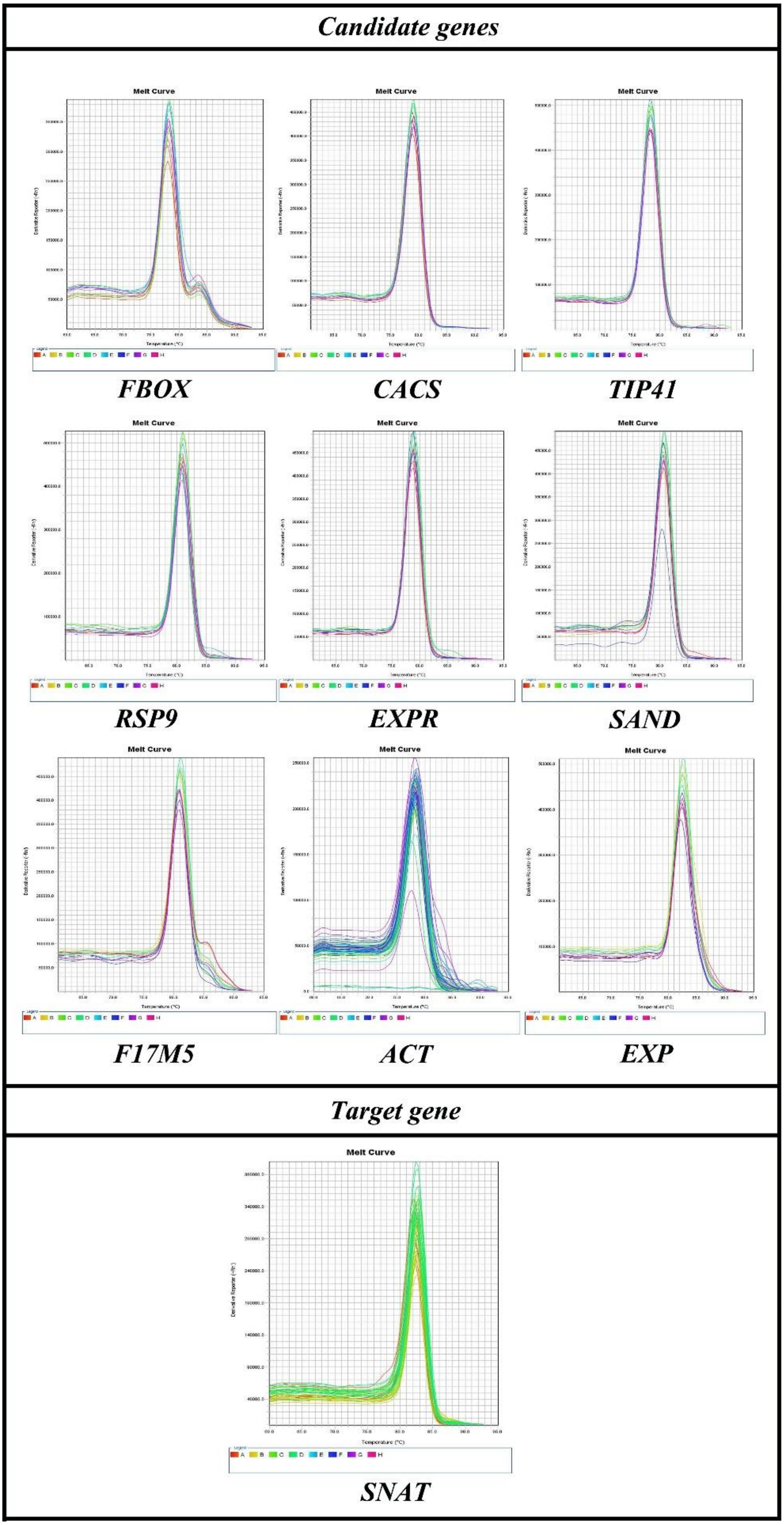

Supplementary Figure-2: Agarose gel electrophoresis-appearance of single amplicon band

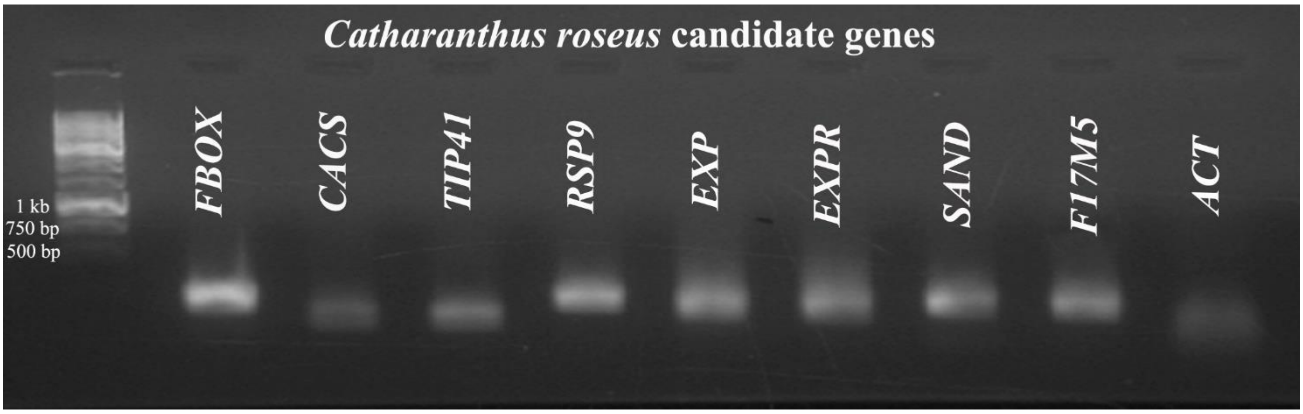

Supplement: Supplementary file 1 — Supplementary Info file [file 41598_2018_20474_MOESM1_ESM.pdf]
